# Treatment with maresin 1, a docosahexaenoic acid-derived pro-resolution lipid, protects skin from inflammation and oxidative stress caused by UVB irradiation

**DOI:** 10.1038/s41598-019-39584-6

**Published:** 2019-02-28

**Authors:** Talita L. C. Cezar, Renata M. Martinez, Camila da Rocha, Cristina P. B. Melo, David L. Vale, Sergio M. Borghi, Victor Fattori, Josiane A. Vignoli, Doumit Camilios-Neto, Marcela M. Baracat, Sandra R. Georgetti, Waldiceu A. Verri, Rubia Casagrande

**Affiliations:** 1 Laboratory of Oxidative Stress and Inflammation, Department of Pharmaceutical Sciences, Londrina State University, Londrina, PR Brazil; 2 Laboratory of Pain, Inflammation, Neuropathy, and Cancer, Department of Pathology, Londrina State University, Londrina, PR Brazil; 3 Department of Biochemistry and Biotechnology, Londrina State University, Londrina, PR Brazil

## Abstract

Acute exposure to UVB irradiation causes skin inflammation and oxidative stress, and long-term exposure to UVB irradiation may lead to carcinogenesis. Our organism has endogenous mechanisms to actively limit inflammation. Maresin 1 (MaR1; 7R,14S-dihydroxy-docosa-4Z,8E,10E,12Z,16Z,19Z-hexaenoic acid) is a pro-resolution lipid mediator derived from the docosahexaenoic acid, which presents anti-inflammatory and pro-resolution effects. However, it remains to be determined if treatment with MaR1 can inhibit inflammatory and oxidative alterations in the skin triggered by UVB. The treatment with MaR1 (0.1–10 ng/mice at −10 min relative to the UVB irradiation protocol) reduced UVB-induced skin edema, neutrophil recruitment (MPO; myeloperoxidase activity, and migration of LysM-eGFP^+^ cells), cytokine production, matrix metalloproteinase-9 activity, keratinocyte apoptosis, epidermal thickening, mast cells counts and degradation of skin collagen in hairless mice. UVB irradiation caused a decrease of GSH (reduced glutathione) levels, activity of the enzyme catalase, ferric reducing ability (FRAP), and ABTS radical scavenging capacity as well as induced lipid hydroperoxide, superoxide anion production, and gp91^phox^ mRNA expression. These parameters that indicate oxidative stress were inhibited by MaR1 treatment. Therefore, these data suggest MaR1 as a promising pharmacological tool in controlling the deleterious effects related to UVB irradiation.

## Introduction

The skin plays a key role as a barrier in innate immunity, thermoregulation, protection against dehydration and release of substances such as melanin that is important against the deleterious effects of ultraviolet radiation (UVR). The skin also presents endogenous antioxidants including reduced glutathione (GSH) and catalase that protect from oxidative stress^1,2^. Despite prominent effect of endogenous protection systems, excessive exposure to UVR can deplete endogenous antioxidants, making the skin susceptible to the deleterious actions of reactive oxygen species (ROS)^3,4^.

The UVR can be divided into UVA (320 to 400 nm), UVB (290 to 320 nm) and UVC (200 to 290 nm). The UVR reaching the surface of the earth is made of up to 95% of UVA and only 5% by UVB^5^. The radiation reaching the skin is in part reflected, refracted and absorbed. The molecule that absorbs this radiation is known as the chromophore. Upon absorbing a photon, the chromophore leaves the idle state and becomes excited, which allows its reaction with molecules in the biological environment leading to formation of photoproducts that will damage the tissue^6^. Both UVA and UVB radiation induce the formation of photoproducts and cause skin damage. Despite the lower percentage of UVB compared to UVA in UVR, UVB is more relevant to photocarcinogenesis than UVA, because it direct damages DNA, RNA, proteins and other cellular components^7,8^.

Understanding the activity of novel compounds with protective activity against the deleterious effects of UVB is an essential step in the development of novel effective therapies^9^. UVB triggers inflammatory and oxidative stress alterations in the skin, thus, drugs targeting these pathological mechanisms are conceivable therapeutic strategies^10–12^. Nevertheless, the progressive knowledge on how inflammation starts and ends, brought into light a novel concept about the resolution phase of inflammation. The resolution of the inflammation is now considered an active process that can also be explored therapeutically^13–15^. Pro-resolution lipid mediators include four classes derived from cell membrane arachidonic acid, eicosapentaenoic acid and docosahexaenoic acid (DHA) which comprise the lipoxins, protectins, resolvins and maresins (MaR)^14^. In general, pro-resolution lipid mediators are by definition known for reducing neutrophil recruitment toward the inflamed tissue and also stimulating non-phlogistic phagocytosis (efferocytosis) by macrophages without impairing bacterial killing^14,16^.

Maresin 1 (MaR1; 7R,14S-dihydroxy-docosa-4Z,8E,10E,12Z,16Z,19Z-hexaenoic acid) has been recently identified as a potent lipid mediator generated endogenously by macrophage enzymes. Interestingly, not only macrophages produce MaR1. For instance, human neutrophil interacts with platelet to further increase endogenous production of MaR1 upon the action of 12-lipoxygenase^17^. In mice, DHA undergoes metabolization by 12/15-lipoxygenases with further enzymatic epoxidation and enzymatic hydrolysis of metabolites to generate bioactive MaR1, while in humans, that conversion is made by 12-lipoxygenase^18,19^. The endogenous production of MaR1 occurs in the resolution phase of the inflammatory process^14^. Evidence suggests that MaR1 acts, at least in part, through the ALXR/FPR2 receptor^20^. Regarding the pharmacological activities of MaR1, this pro-resolution lipid lowers the epithelial bronchial cell production of interleukin (IL)-6 and IL-8 by a mechanism related to the blockade of protein kinase Cε (PKCε) and PKCα activities in a model of dust extract-induced allergic inflammation^21^. Treatment with MaR1 also reduced 2,4,6-trinitrobenzenesulfonic acid- and dextran sulfate sodium-induced inflammatory bowel disease by reducing the production of cytokines such as tumor necrosis factor-α (TNFα), IL-1β, IL-6 and interferon-γ (IFNγ) depending on the stage of the disease. These effects were attributed to the inhibition of nuclear factor κB (NFκB) alongside with a switch of macrophages to a M2 anti-inflammatory/alternative activation phenotype^22^. MaR1 reduced lipopolysaccharide (LPS)-induced innate immune pulmonary inflammation by inhibiting the production of cytokines such as TNFα, IL-1β, IL-6 and the chemokines keratinocyte-derived chemokine (KC), monocyte chemoattractant protein (MCP)-5, macrophage inflammatory protein (MIP)-1α and MIP-1γ. The decrease on cytokine production resulted in lowering the expression of adhesion molecules including intercellular adhesion molecule (ICAM)-1, P-selectin and CD24, and consequently lung neutrophil recruitment/myeloperoxidase (MPO) activity^23^. MaR1 also enhances the efferocytosis of apoptotic neutrophils by macrophages, limits the influx of inflammatory cells and induces non-phlogistic macrophages^18,19^. Moreover, MaR1 reduces vascular inflammation by interfering with inflammatory signaling in endothelial and vascular smooth muscle cells of human saphenous vein^24^. In addition to these anti-inflammatory and pro-resolution effects, MaR1 also acts as an analgesic by inhibiting transient receptor potential cation channel subfamily V member 1 (TRPV1)-induced neuronal currents and reducing chemotherapy-induced neuropathic pain^25^. The prominent regulatory properties of MaR1 in inflammation have raised the question on whether this endogenous lipid could function as an exogenous biological anti-inflammatory/pro-resolution active principle in medication. In the present study, the anti-inflammatory effect of treatment with MaR1 was investigated in a model of UVB-triggered development of inflammatory and ROS alterations in the skin of mice.

## Results

### MaR1 inhibits the development of skin edema, MPO activity and the recruitment of LysM-eGFP^+^ cells triggered by UVB irradiation

**Figure 1:**
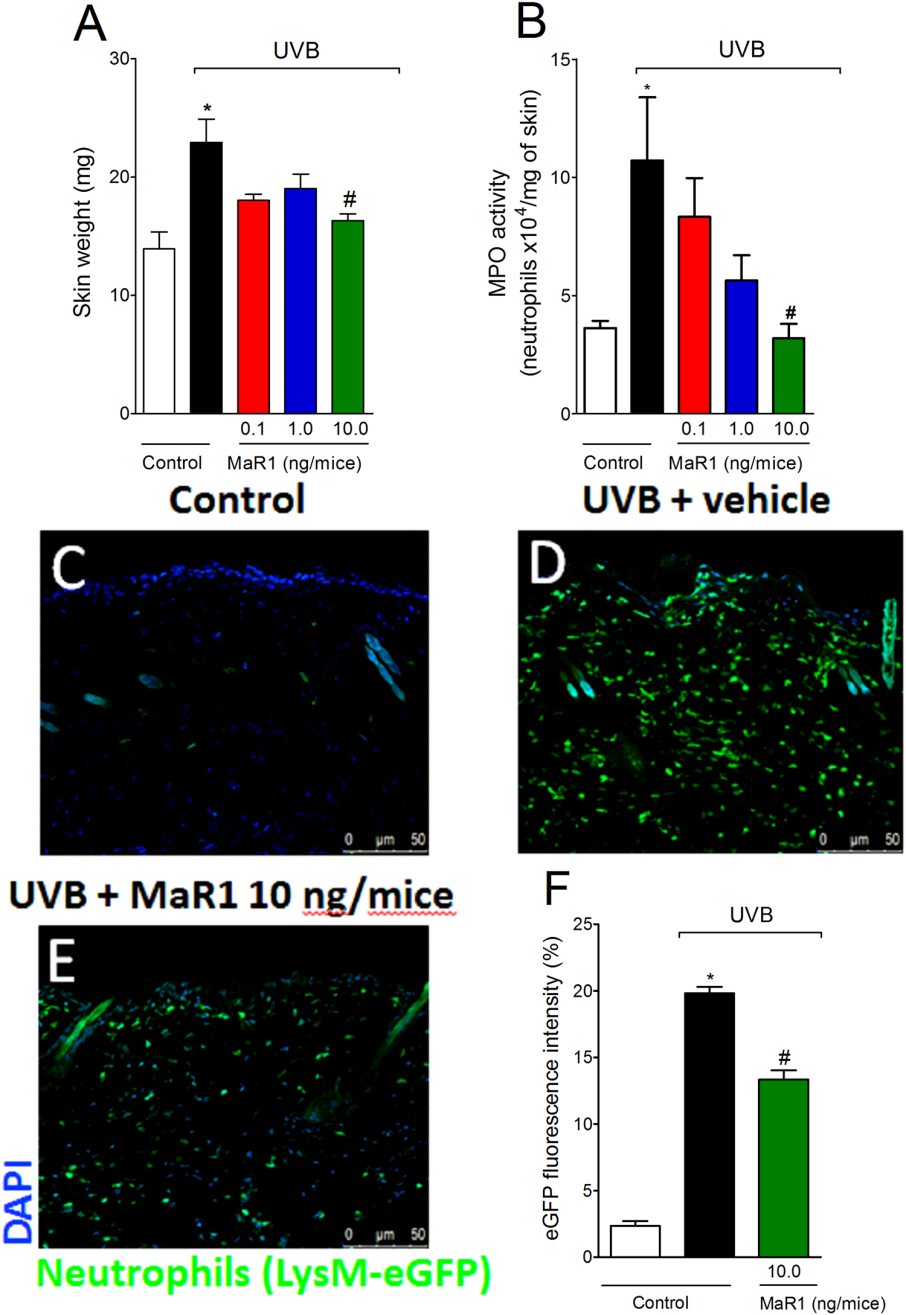
MaR1 reduces the development of skin edema, MPO activity and the recruitment of LysM-eGFP+ cells triggered by UVB irradiation. The skin edema (**A**), MPO activity (**B**) and LysM-eGFP+ cells recruitment (**C**–**F**) were determined in samples dissected 12 h after the radiation. Results are presented as tissue weight in milligrams for skin edema and as neutrophils x10^4^ per milligram of tissue for MPO activity. Original magnification 20x (images C–E); 50 μm. *p < 0.05 compared to non-irradiated group and ^#^p < 0.05 compared to irradiated vehicle-treated group.

Exposure to UVB induces an acute response in the skin that includes formation of edema and leukocyte recruitment^8,26^. Based on that, we first investigated the effect of MaR1 (0.1–10 ng) on two inflammation parameters: skin edema, a classical sign of inflammation and neutrophil recruitment, an important cellular event during inflammation. UVB irradiation triggered both skin edema (Fig. 1A) and MPO activity (Fig. 1B) compared to negative control group (non-irradiated group), which were amenable by the dose of 10 ng of MaR1. To determine whether the reduction of MPO activity was related to lowering the counts of recruited immune cells, we performed fluorescence analysis using LysM-eGFP mice. Corroborating the MPO data, we observed an increase of LysM-eGFP^+^ cells in the skin tissue after UVB irradiation and treatment with the 10 ng-dose of MaR1 inhibited this cellular recruitment (Fig. 1C–F). Method differences might explain the discrepancies on the intensity of effect of MaR1 on skin inflammation. The MPO activity is an enzymatic assay and indicates indirectly the presence of neutrophils, but also reflects the activity of MPO in these cells^11,27^. The fluorescence data used a reporter mouse strain (LysM-eGFP) to determine cellular presence and not enzyme activity. Altogether, these data demonstrate that MaR1 reduces UVB irradiation-induced skin inflammation.

### MaR1 inhibits UVB irradiation-induced apoptosis of keratinocytes

**Figure 2:**
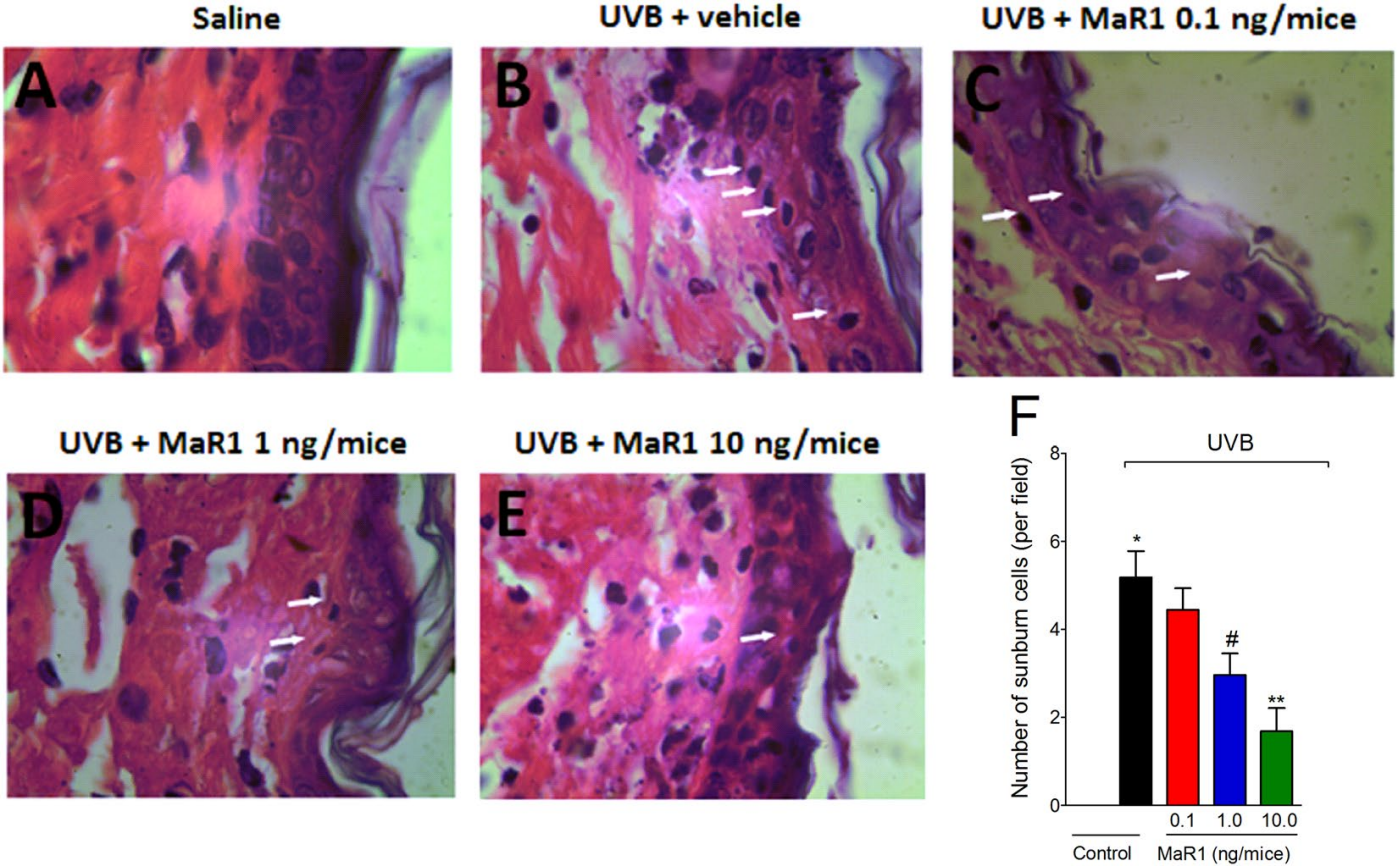
MaR1 reduced UVB irradiation-induced sunburn cells formation. The number of sunburn cells was determined in samples dissected 12 h after the radiation and stained with H&E. Representative images of non-irradiated control (**A**), irradiated treated with vehicle (**B**), irradiated treated with 0.1 ng of MaR1 (**C**), irradiated treated with 1 ng of MaR1 (**D**), and irradiated treated with 10 ng of MaR1 (**E**) groups are presented. Quantitative analysis of sunburn cells in experimental groups is presented per field in panel F. Original magnification 100x; 100 μm. *p < 0.05 compared to non-irradiated group; ^#^p < 0.05 compared to irradiated vehicle-treated group; and **p < 0.05 compared to irradiated vehicle- and 0.1 ng of MaR1-treated groups.

After UVB radiation, keratinocytes undergo apoptosis (sunburn cells)^28^. Histologically, sunburn cells present altered morphology as observed by chromatin condensation and eosinophilic cytoplasm^28^. UVB irradiation induced an increase in the number of sunburn cells compared to negative control group (Fig. 2A and B). On the other hand, MaR1 reduced the number of sunburn cells in a dose-dependent manner with significant effects with 1 and 10 ng (Fig. 2C–F). MaR1 at 10 ng presented a statistically different effect compared to the 0.1 ng dose (Fig. 2F). These data indicate a protective effect of MaR1 upon UVB irradiation-induced keratinocytes apoptosis.

### MaR1 inhibits UVB irradiation-induced epidermal thickening

**Figure 3:**
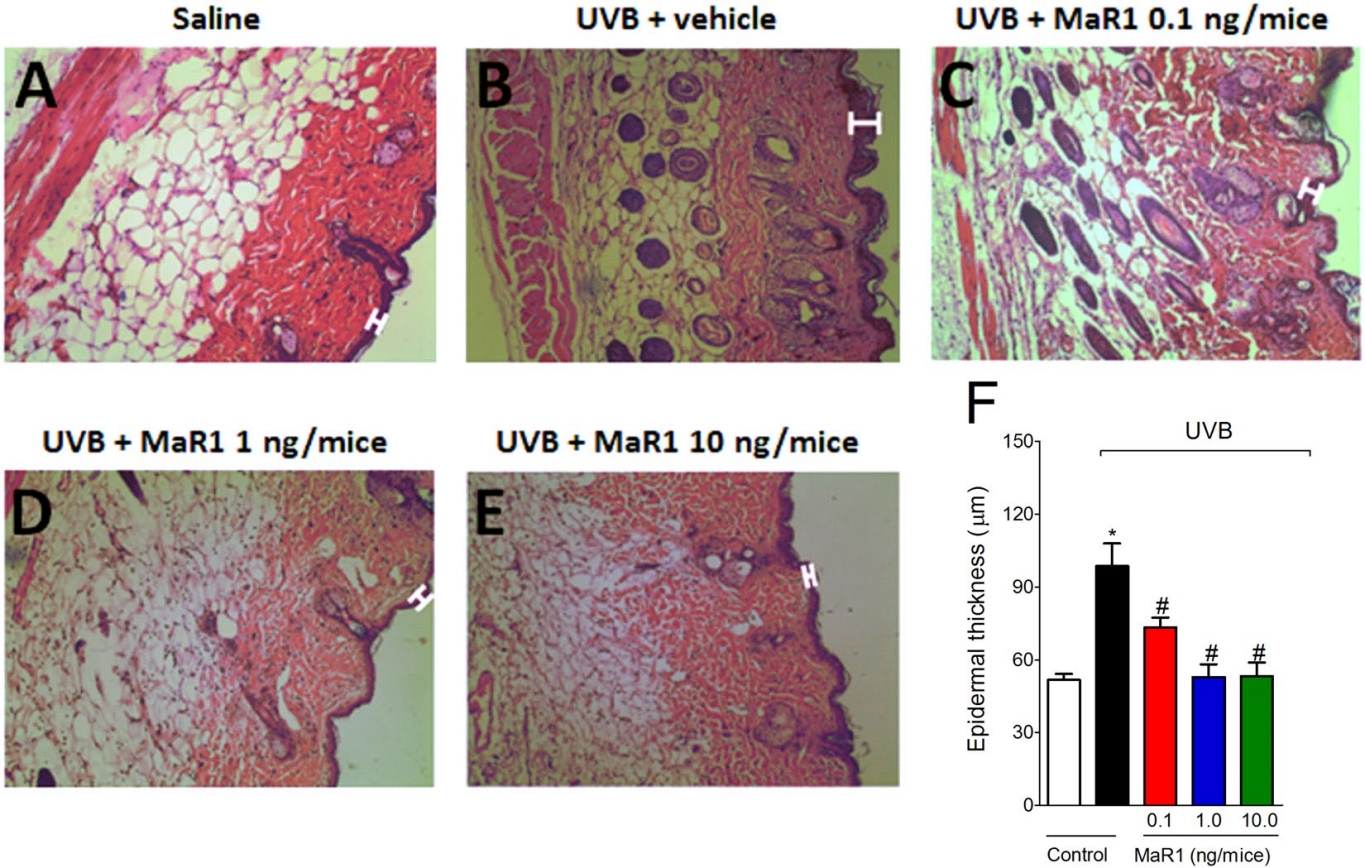
MaR1 reduced UVB irradiation-induced epidermal thickening. The epidermal thickness was determined in samples dissected 12 h after the radiation and stained with H&E. Representative images of non-irradiated control (**A**), irradiated treated with vehicle (**B**), irradiated treated with 0.1 ng of MaR1 (**C**), irradiated treated with 1 ng of MaR1 (**D**), and irradiated treated with 10 ng of MaR1 (**E**) groups are presented. Epidermal thickness of experimental groups is presented in μm in panel F. Original magnification 20x; 100 μm. *p < 0.05 compared to non-irradiated group and ^#^p < 0.05 compared to irradiated vehicle-treated group.

UVB irradiation also causes epidermal thickening due to edema, cellular infiltration and cellular proliferation^29^. UVB irradiation induced significant epidermal thickening in comparison to the negative control group (Fig. 3A and B), which was inhibited by all doses of MaR1 tested (0.1, 1 and 10 ng; Fig. 3C–F). The doses of 1 and 10 ng abolished the epidermal thickening caused by UVB (Fig. 3F). This result demonstrates that MaR1 reduces the deleterious impact imposed by UVB radiation to the skin.

### MaR1 inhibits UVB irradiation-induced increase of mast cell counts

**Figure 4:**
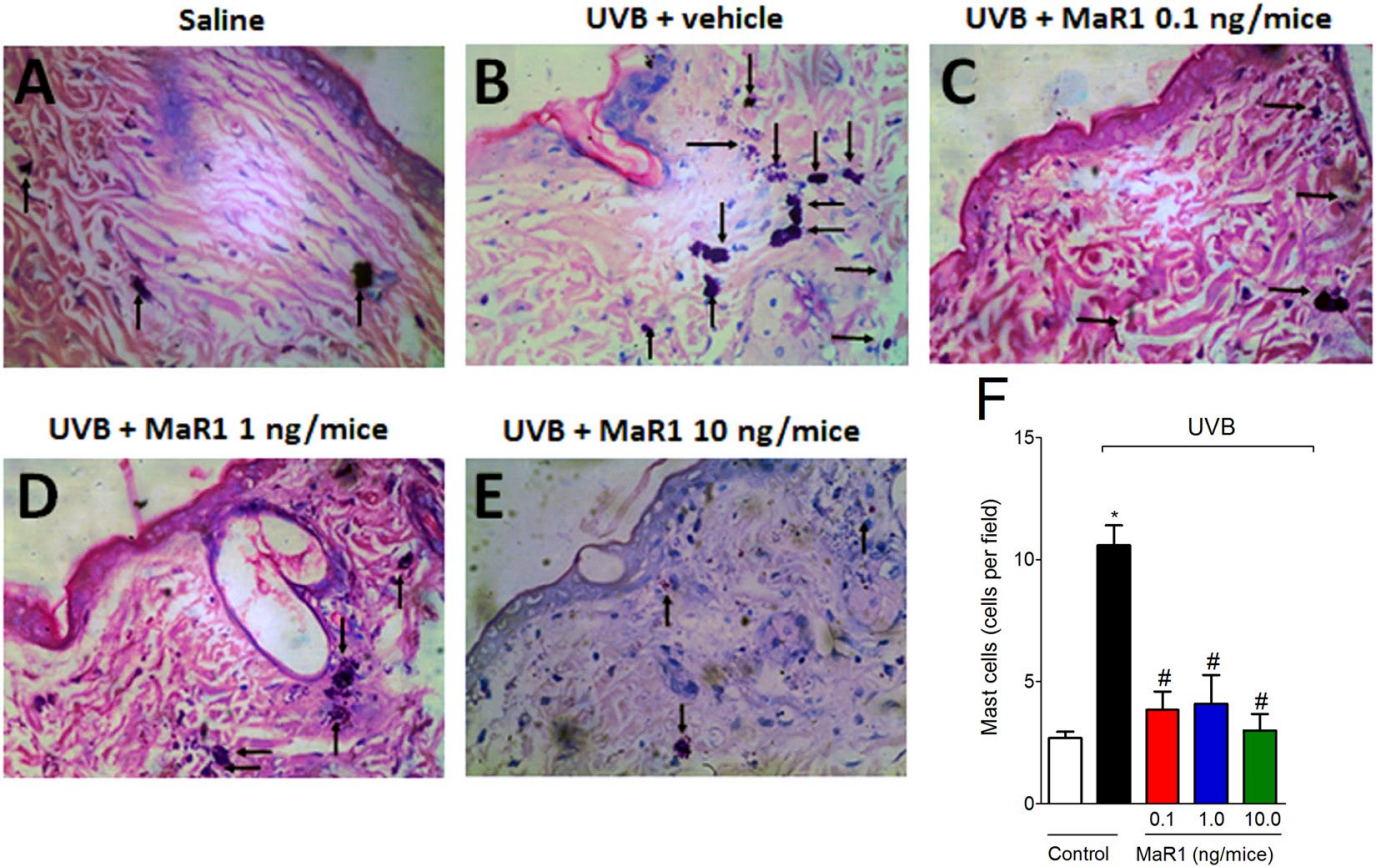
MaR1 reduced UVB irradiation-induced mast cells counts. The proliferation of mast cells was determined in samples dissected 12 h after the radiation and stained with toluidine blue. Representative images of non-irradiated control (**A**), irradiated treated with vehicle (**B**), irradiated treated with 0.1 ng of MaR1 (**C**), irradiated treated with 1 ng of MaR1 (**D**), and irradiated treated with 10 ng of MaR1 (**E**) groups are presented. Mast cells count of experimental groups is presented per field in panel F. Original magnification 40x; 100 μm. *p < 0.05 compared to non-irradiated group and ^#^p < 0.05 compared to irradiated vehicle-treated group.

After UVB irradiation, mast cells secrete mediators that trigger inflammation and recruit other leukocytes such as neutrophils^30^. UVB irradiation induced significant increase of mast cells compared to basal conditions of negative control group (Fig. 4A and B). All doses of MaR1 reduced the mast cell counts in the skin (Fig. 4C–F). These data indicate that besides inhibiting neutrophils recruitment (MPO and LysM-eGFP data), MaR1 also ameliorates UVB irradiation-induced inflammation by effectively reducing the numbers of mast cells in the skin.

### MaR1 inhibits UVB irradiation-induced MMP-9 activity and collagen fibers degradation

**Figure 5:**
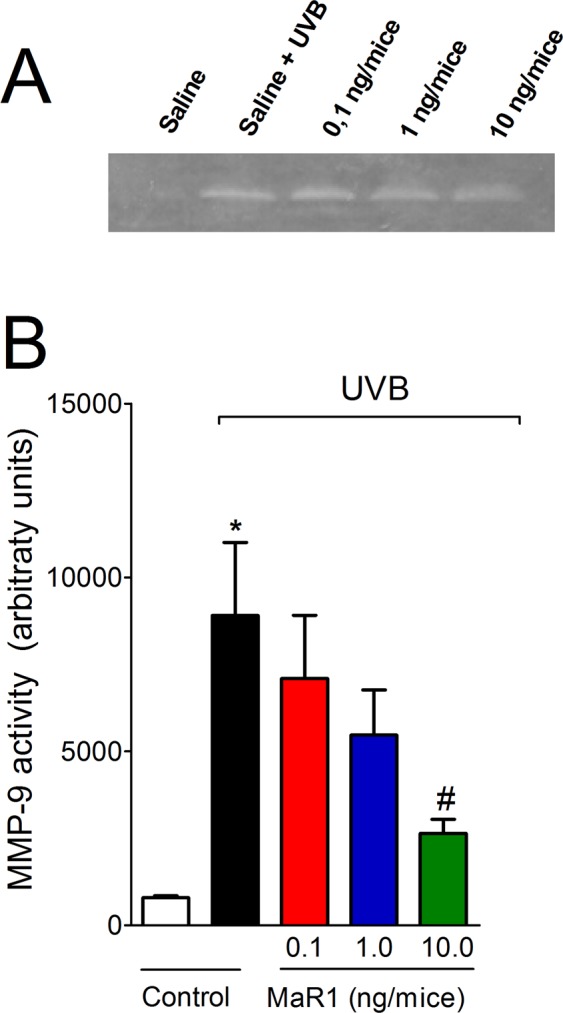
MaR1 inhibited UVB irradiation-induced MMP-9 activity in the skin. MMP-9 activity was determined in samples dissected 12 h after the radiation. Image of gelatin zymography is presented in panel A and skin MMP-9 activity in panel B. Uncropped full-length gels are presented in Supplementary Fig. S1. MMP-9 MW 92 kDa. Results are presented as arbitrary units per sample for MMP-9 activity. *p < 0.05 compared to non-irradiated group and ^#^p < 0.05 compared to irradiated vehicle-treated group.

**Figure 6:**
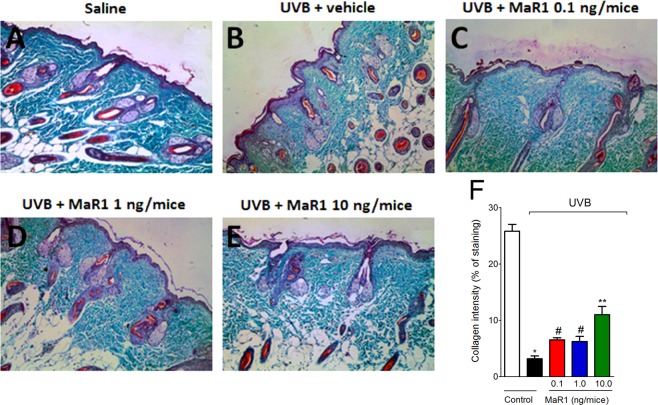
MaR1 inhibited UVB irradiation-induced collagen degradation in the skin. Degradation of collagen was determined in samples dissected 12 h after the radiation and stained with Masson’s trichrome. Representative images of non-irradiated control (**A**), irradiated treated with vehicle (**B**), irradiated treated with 0.1 ng of MaR1 (**C**), irradiated treated with 1 ng of MaR1 (**D**), and irradiated treated with 10 ng of MaR1 (**E**) groups are presented. Quantitative analysis of collagen degradation of experimental groups is presented as percentage of staining in panel F. Original magnification 20x; 100 μm. *p < 0.05 compared to non-irradiated group; ^#^p < 0.05 compared to irradiated vehicle-treated group; and **p < 0.05 compared to irradiated vehicle- and 0.1 ng of MaR1-treated groups.

Photoaging and severe skin damage are the most common symptoms associated with continuous exposure to UVB. Those processes are mediated by metalloproteinases, which are enzymes expressed by epidermal keratinocytes and dermal fibroblasts. These enzymes degrade collagen and other extracellular matrix proteins^30^. We observed that MMP-9 activity was reduced by MaR1 only at the dose of 10 ng (Fig. 5A and B). Given the deleterious effect of MMP-9 enzymatic activity on collagen fibers, we next performed Masson’s trichome staining, because it allows the quantification of collagen. Treatment with MaR1 at all doses reduced the degradation of skin collagen, as observed by the preservation on the blue/green color in the Masson’s trichrome staining. Therefore, the enzymatic activity assay (MMP-9 assay) and tissue staining (Masson’s trichome) results corroborate each other (Fig. 6A–F). These data demonstrate that MaR1 reduces dermal connective tissue damage.

### MaR1 inhibits UVB irradiation-triggered production of cytokines and gp91^phox^ mRNA expression

**Figure 7:**
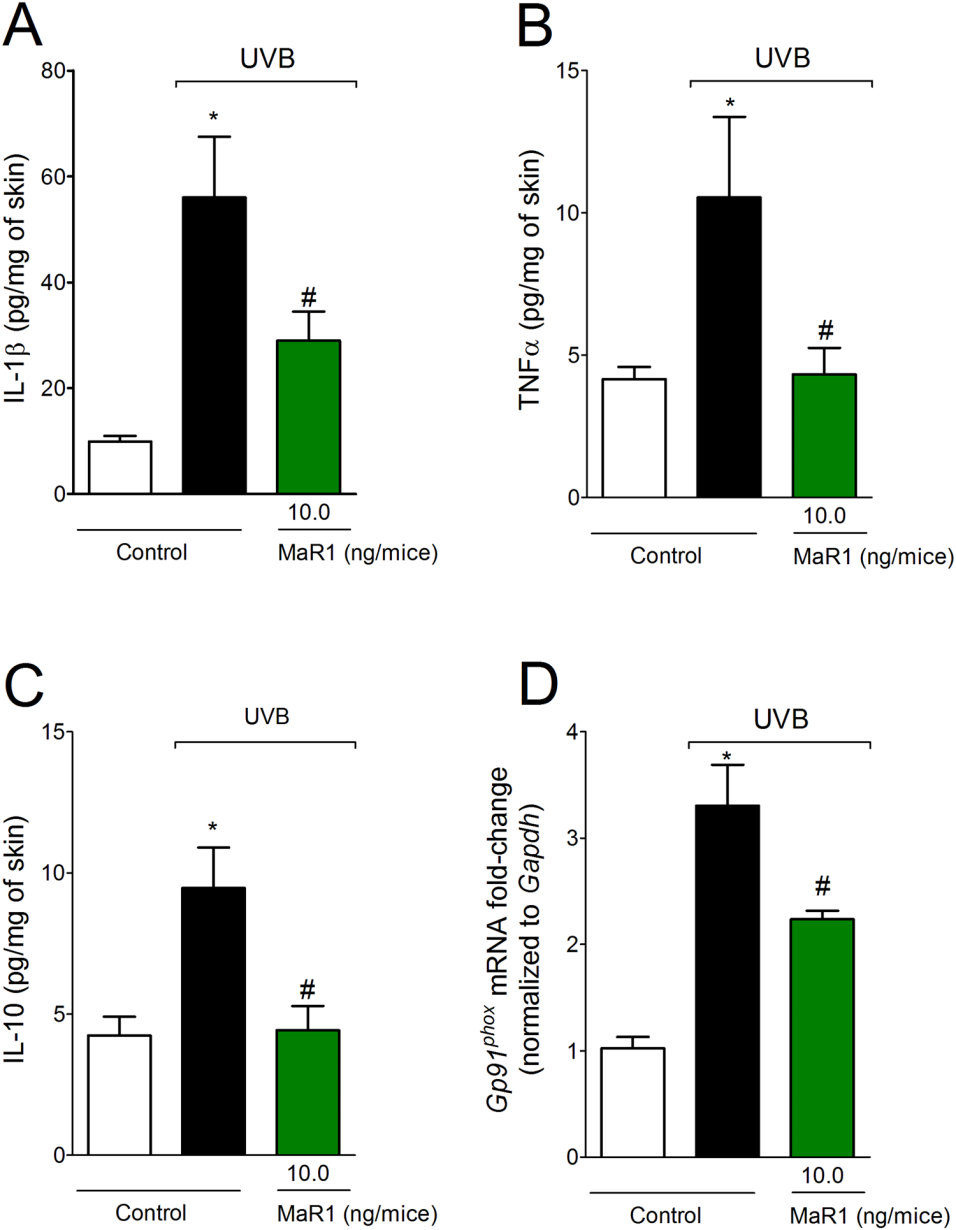
MaR1 inhibits the production of cytokines and gp91phox mRNA expression in the skin triggered by UVB irradiation. Skin samples were dissected 4 h after the radiation to determine the levels of IL-1β (**A**), TNF-α (**B**), and IL-10 (**C**) by ELISA. Results are presented as picograms per milligrams of tissue. Skin samples were dissected 4 h after the radiation to determine gp91^phox^ mRNA expression (**D**) by RT-qPCR. *p < 0.05 compared to non-irradiated group and ^#^p < 0.05 compared to irradiated vehicle-treated group.

The cytokines IL-1β, TNFα and IL-10 are produced after excessive exposure to UVB radiation^11,12,28,31^. In the light of the pro-inflammatory contribution of IL-1β and TNFα and the role of IL-10 in limiting inflammation, the putative effect of MaR1 on cytokine production was addressed. We observed that the increase on cytokine levels after exposure to UVB was counteracted by MaR1 at 10 ng (Fig. 7A–C). As a consequence of the inflammation caused by UVB, recruited cells such as neutrophils and macrophages produce large amounts of reactive oxygen species (ROS) such as superoxide anion^8,29^. Superoxide anion formation depends on gp91^phox^ (also known as NOX2), a subunit of nicotinamide adenine dinucleotide phosphate (NADPH) oxidase^11^. Thus, it was next addressed whether MaR1 reduces gp91^phox^ mRNA expression. We observed that MaR1 inhibited gp91^phox^ mRNA expression at the dose of 10 ng (Fig. 7D). These results demonstrate that MaR1 targets cytokines and oxidative metabolism to reduce UVB-irradiation induced inflammatory injury.

### MaR1 inhibits UVB irradiation-induced oxidative stress

**Figure 8:**
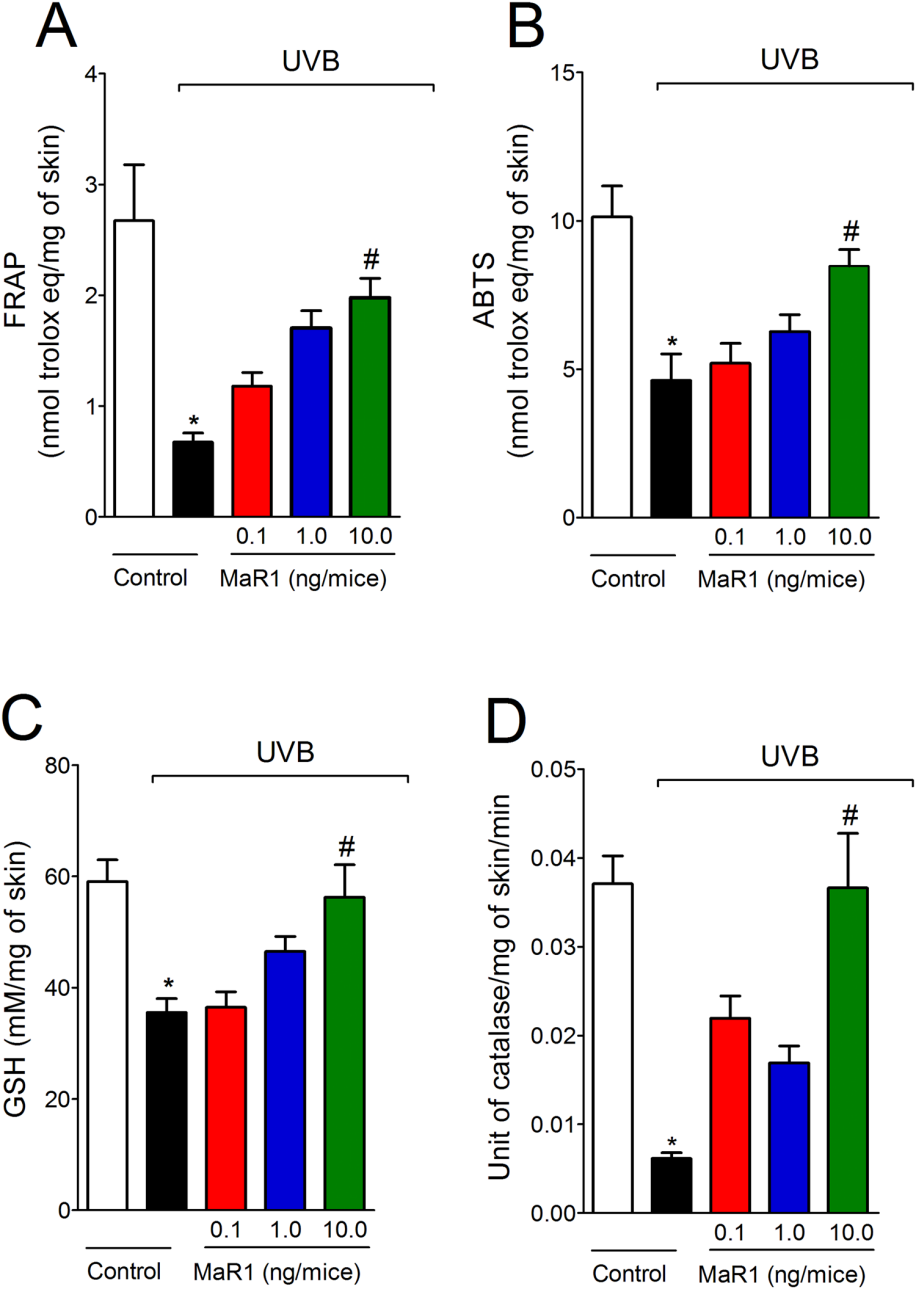
MaR1 inhibited UVB irradiation-induced decrease of skin antioxidant capacity. Total antioxidant capacity (FRAP [**A**], ABTS [**B**]) and GSH levels (**C**) were determined in samples dissected 12 h after the radiation. For the catalase assay (**D**), samples were dissected 2 h after the radiation. Results are presented as nanomol of trolox per milligrams of tissue for FRAP and ABTS assays, as millimoles per milligrams of tissue for GSH assay and as unit of catalase per milligrams of tissue per minutes for catalase assay. *p < 0.05 compared to non-irradiated group and ^#^p < 0.05 compared to irradiated vehicle-treated group.

**Figure 9:**
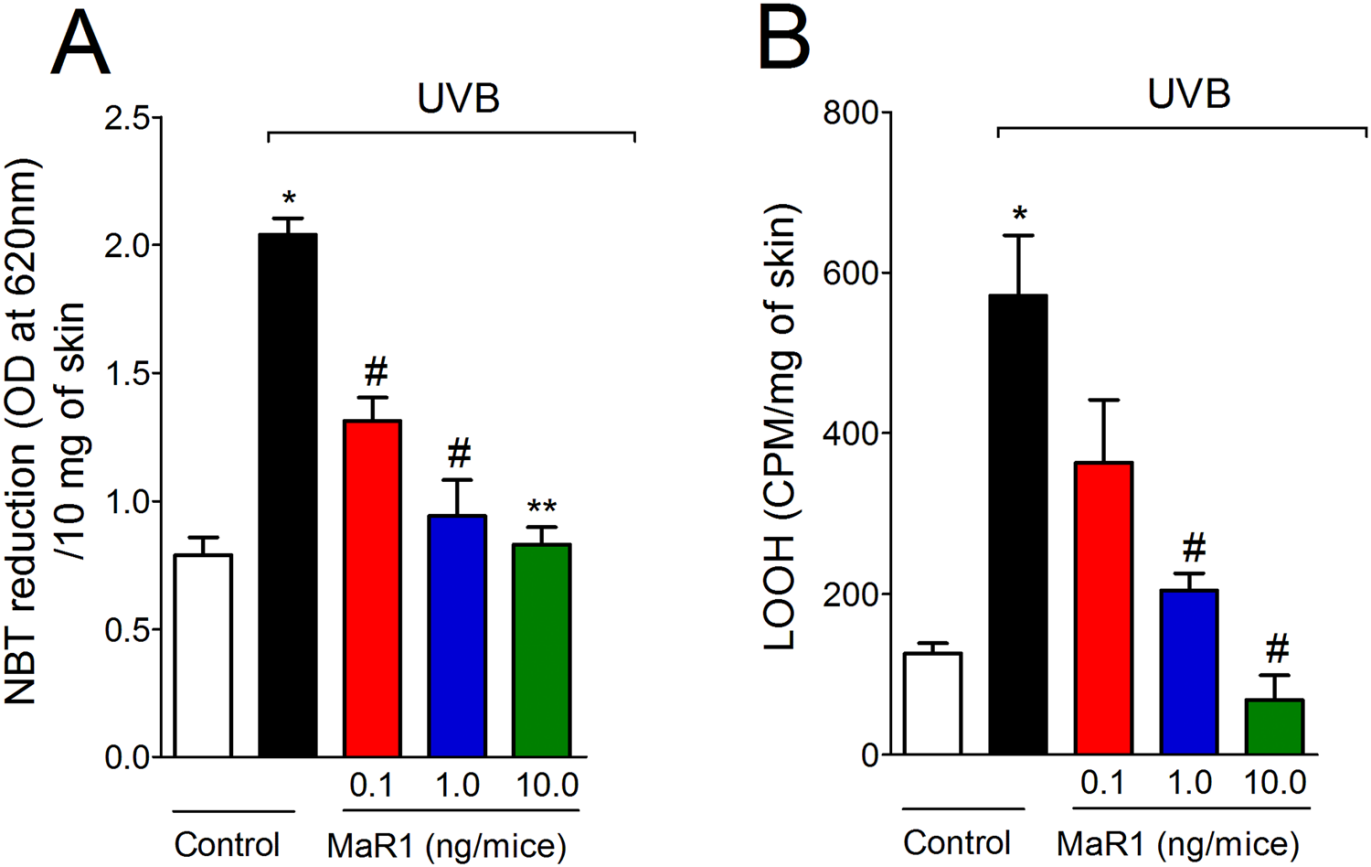
MaR1 inhibited UVB irradiation-induced superoxide anion production and lipid peroxidation. Superoxide anion production (NBT reduction) (**A**) and lipid peroxidation (LOOH) (**B**) were determined in samples dissected 4 h and 2 h after the radiation, respectively. Results are presented as OD at 620 nm per 10 milligrams of tissue and as CPM per milligrams of tissue, respectively. *p < 0.05 compared to non-irradiated group; ^#^p < 0.05 compared to irradiated vehicle-treated group; and **p < 0.05 compared to irradiated vehicle and 0.1 ng of MaR1-treated groups.

Given UVB irradiation decreases antioxidant defenses as well as increases other oxidative stress markers in the skin^11,12,32,33^, we next evaluated the effect of MaR1 on UVB-induced oxidative stress. UVB irradiation depleted the antioxidant capacity of the skin as observed by reduced activity/levels in the FRAP assay (Fig. 8A), ABTS assay (Fig. 8B), GSH quantitation (Fig. 8C) and catalase activity assay (Fig. 8D). In addition to reducing antioxidant defenses (Fig. 8A–D), UVB irradiation increased superoxide anion production (Fig. 9A) and lipid peroxidation (Fig. 9B), which are markers of oxidative stress. MaR1 at 10 ng restored the skin antioxidant capacity as observed in the FRAP, ABTS, GSH and catalase assays (Fig. 8). Furthermore, all three doses of MaR1 inhibited superoxide anion production (the dose of 10 ng also present significant effect compared to the lower dose of 0.1 ng; Fig. 9A), while only MaR1 at 1 and 10 ng inhibited lipid peroxidation (Fig. 9B). Thus, the antioxidant effects of MaR1 contributed to the attenuation of the skin damages caused by UVB irradiation.

## Discussion

A current concept is that pro-resolution lipid mediators actively reduce inflammation. Imbalance on pro-resolution lipid mediator profile can be found in skin diseases such as psoriasis. Interestingly, despite the presence of pro-resolution lipid mediators and their precursors in the skin of patients with psoriasis, it is also possible to observe a high increase on omega-6 fatty acid-oxidized derivatives, which shift the response to a pro-inflammatory profile^34,35^. Therefore, this imbalance can favor the development of skin inflammation and indicates that treatment with pro-resolution lipid mediators may work as a therapeutic approach to treat skin diseases. In fact, we have recently demonstrated that treatment with Lipoxin A_4_ and Resolvin D1 (RvD1), which are pro-resolution lipid molecules derived from arachidonic acid and DHA, respectively, reduce UVB irradiation-induced skin damages^11,36^. However, to our knowledge, the present study shows for the first time that treatment with MaR1 efficiently reduces the deleterious impact of UVB-induced skin injury through the inhibition of inflammation and oxidative stress.

MaR1 reduced clinical signs of inflammation and essential pathophysiological inflammatory parameters in the UVB-induced skin injury model. MaR1 (10 ng per mouse) inhibited skin edema, neutrophils/macrophages recruitment (MPO and LysM-eGFP data), number of sunburn cells, epidermal thickening and mast cell counts triggered by UVB exposure in a dose-dependent manner. These results are in line with the inhibitory effect of MaR1 on neutrophil chemotaxis and stimulation of efferocytosis by macrophages^25,37^. Furthermore, human neutrophil interacts with platelet to increase endogenous production of MaR1, which might contribute to limit neutrophil recruitment toward tissue^17^. UVB also induces the apoptosis of keratinocytes, which can be observed as sunburn cells^28^. In a murine model of cancer, RvD1, RvD2, and RvE1 reduce apoptotic tumor cell debris-stimulation of tumor growth, indicating that enhancing the clearance of apoptotic cells and debris can reduce inflammation^38^. Herein we show that MaR1 inhibited keratinocyte apoptosis, thus, reducing the number of sunburn cells. Therefore, concurrent data reveal the inhibition of UVB-induced skin inflammation by MaR1.

Keratinocytes contribute to the initiation of the inflammatory response upon UVB irradiation by producing cytokines and chemokines^28^. These mediators induce vascular permeability, edema and recruitment of inflammatory cells (e.g. neutrophils)^31,39^. Tissue resident cells, such as mast cells also release histamine, TNFα, and IL-1β to stimulate the expression of metalloproteinase (especially MMP-9) in skin keratinocytes and fibroblasts. MMP-9 degrades collagen causing the skin photoaging^40–42^. In fact, considering the vulnerability of the skin to UV irradiation, MMP-9 is strongly linked to pathophysiology of tumors^43^. Interestingly, the treatment with MaR1 reduced mast cells count as well as inhibited the MMP-9 activity and the production of TNFα and IL-1β, indicating that MaR1 targets these physiopathological mechanisms to reduce UVB-induced skin inflammation and injury. TNFα also contributes to the generation of sunburn cells^28,44^. Thus, the inhibition of TNFα by MaR1 is in accordance with the reduced number of sunburn cells detected in our model. Additionally, both IL-1β and TNFα are edematogenic^45^ and induce neutrophil recruitment^46^. Therefore, it is likely that MaR1 inhibition of TNF-α and IL-1β might have accounted to reduce edema, keratinocytes apoptosis, mast cell count, neutrophil recruitment, MMP-9 activity and collagen degradation induced by UVB irradiation. IL-10 is an anti-inflammatory cytokine that is co-released with pro-inflammatory cytokines to limit inflammation^47^. The present results demonstrated that MaR1 inhibited UVB-induced IL-10 production, which indicates that enhancing IL-10 levels is not a therapeutic mechanism of MaR1 in UVB irradiation-triggered inflammation. It is also possible that as MaR1 reduced the release of pro-inflammatory cytokines, the co-release of IL-10 was also reduced^48^. Collectively, these data corroborate other studies using different models and demonstrate that inhibiting cytokine production is a consistent mechanism of action of MaR1^22,23^. Interestingly, in a model using human bronchial epithelial cells line (BEAS-2B) and mouse lung slices stimulated with hog confinement facility-derived organic dust extract (HDE), MaR1 inhibited the release of pro-inflammatory cytokines and chemokines (TNF-α, IL-6, and CXCL1). In terms of mechanism, the HDE extract induced DNA biding activities of NFκB, AP-1 (activator protein 1), SP-1 (trans-acting transcription factor 1), and SRE (serum response element) as determined using luciferase reporter assays. However, MaR1 only inhibited the SRE DNA binding activity^21^, which demonstrated that MaR1 mechanisms may depend on inflammatory stimulus context and supports the importance of investigating MaR1 activities and mechanisms in every condition it may be useful.

Excessive absorption of UV irradiation can lead to the formation of molecular oxygen (O_2_) increasing ROS production that trigger NFκB activation and cytokine production^6,33,49^. UVB also induces H_2_O_2_ directly and indirectly though O_2_^•−^ production^4^, and increases cyclooxygenase 2 (COX-2) activity resulting in pro-inflammatory prostaglandin production^50^. On the other hand, the activity of GSH and catalase are endogenous skin mechanisms to maintain its integrity. The first one chelates metal ions forming inert complexes preventing the oxidation process, while catalase converts H_2_O_2_ into H_2_O and O_2_ that prevents hydroxyl radicals (^•^OH) formation. However, UVB irradiation-induced ROS production overwhelms the endogenous antioxidant systems resulting in tissue lesion^33^. Pro-resolution lipid mediators reduce oxidative stress *in vitro* as evidenced by two recent studies showing RvD1^51^ and RvE1^52^ reduce ROS production in macrophages. Interestingly, despite reducing ROS production, pro-resolution lipid mediators are not immunosuppressive. For instance, RvD1, RvD5, and PD1 lower the requirement of antibiotics during *E*. *coli* infection since these pro-resolution lipids enhance bacterial killing by neutrophils and macrophages^16^. This effect could be particularly interesting since disruption of skin barrier can increase susceptibility to infections^53^. In the present study, MaR1 restored the levels of GSH and catalase activity as well as reduced O_2_^•−^ production and gp91^phox^ (NOX2) mRNA expression. These data are consistent with the effect of MaR1 on attenuating TNFα-induced ROS production, expression of NOX2, adhesion molecules and NFκB activation in vascular smooth muscle and endothelial cells^24^. The reduction of gp91^phox^ mRNA expression observed upon treatment with MaR1 could be related to the lower amount of recruited immune cells in the UVB irradiation model of skin sterile inflammation and does not directly indicates immunosuppression, which was not addressed in the present work. We also observed that MaR1 restored the ability of the skin to reduce the ion Fe^+3^ to Fe^+2^ and the ability to scavenge the ABTS^+^ radical. Notably, oxidative stress is also involved in sunburn cells formation through mechanisms that lead to cytochrome C release after mitochondrial injury and modulation of p53 activity^54,55^. Consistent with these concepts, aldose reductase (an enzyme transcriptionally regulated during cellular response against oxidative insults and toxic aldehydes) diminishes human keratinocytes (HaCaT cells) apoptosis after UVB exposure. These effects were attributed to be dependent on p53 regulation, possibly through a suppression of ROS generation after UVB irradiation^54^. Therefore, besides inhibition of TNFα, counteracting oxidative damage is also a contributing mechanism of MaR1 to reduce the formation of sunburn cells.

Summing up, intraperitoneal administration of the pro-resolution lipid mediator MaR1 protected the skin against inflammatory and oxidative alterations triggered by irradiation with UVB light. MaR1 diminished the following UVB skin physiopathological alterations: edema, neutrophil recruitment, the number of sunburn cells, epidermal thickening, mast cell counts, collagen degradation and MMP-9 activity. The inhibition of cytokine and ROS production explained the therapeutic actions of MaR1. Importantly, MaR1 presented a dose-dependent effect indicating a rational dosing that favors clinical development.

## Materials and Methods

### Drugs and reagents

Reagents from Sigma-Aldrich (St. Louis, MO, USA) were GSH (reduced glutathione), brilliant blue R, DTNB (5,5′- dithiobis[2-nitrobenzoic acid]), N-ethylmaleimide, HTAB (hexadecyltrimethylammonium bromide), ABTS (phenylmethanesulfonyl fluoride, 2,2′-azino-bis[3-ethylbenzothiazoline-6-sulfonic acid]), *o*-dianisidine dihydrochloride, TPTZ (2,4,6-Tris(2-pyridyl)-*s*-triazine), NBT (nitroblue tetrazolium), DMSO (dimethylsulfoxide) and bisacrylamide. Reagent from Bio-Rad Laboratories (Hercules, CA, USA) was Precision Plus Protein^TM^ Kaleidoscope^TM^ Prestained Protein Standards #1610375. Reagent from Cayman Chemical (Ann Arbor, Michigan, USA) was MaR1 (maresin 1; 7R,14S-dihydroxy-docosa-4Z,8E,10E,12Z,16Z,19Z-hexaenoic acid). Reagent from Acros (Pittsburgh, PA, USA) was tert-butyl hydroperoxide. Reagents from Amresco (Solon, OH, USA) were Tris and xylene cyanol. ELISA commercial kits from eBioscience (San Diego, CA, USA) were used for determination of cytokine production. Reagents from Thermo Fisher Scientific (Waltham, MA, USA) were sodium dodecyl sulfate (SDS) (Superscrip III) and acrylamide. All other reagents used were from pharmaceutical grade.

### Animals

The experiments were performed in hairless mice (HRS/J) or LysM-eGFP C57BL/6 background mice weighing 20–30 g, sex matched and obtained from the Londrina State University (UEL), Paraná, Brazil. Housing was under pathogen-free conditions in cages with individual ventilation in a rack system designed for mouse with regular shaving bedding and free access to water and food, with light/dark cycle of 12/12 h, exhausted air and controlled temperature (22 ± 2 °C). The Ethics Committee for Animal Use (CEUA/UEL, process number 1447.2015.10) approved all procedures of this study. All methods were performed in accordance with the relevant guidelines and regulations. All efforts were made in order to minimize the number of animals used and their suffering. Euthanasia at the end of experiments involved the sequential procedures of anesthesia with isoflurane 5% (Abbott Park, IL, USA), terminal killing by cervical dislocation and decapitation. Euthanasia was always performed during the light cycle. The mice were continuously monitored regarding welfare-related assessment before, during and after the experiments.

### General experimental procedures

Hairless mice (n = 6 mice per group per experiment) or LysM-eGFP mice (n = 5 mice per group per experiment) were randomly assigned in three different groups: non-irradiated, vehicle-treated irradiated group, and MaR1-treated irradiated group. The stock solution contained 10 ng/μL of MaR1 in 100% ethanol and was kept in a −80 °C freezer until use. During the preparation of the doses for the treatments, caution was taken to avoid exposure of MaR1 to air. The doses of MaR1 were chosen based on previous reports^18,22,56^. Mice were treated with MaR1 (0.1, 1, or 10 ng/mice, diluted in sterile saline, intraperitoneally, 200 µL [1 μL of ethanol plus 199 μL of saline]) 10 min before the beginning of UVB irradiation. Based on the dose-dependent results of MPO activity, we used the dose of 10 ng/mice for experiments involving LysM-eGFP mice. Samples of skin were dissected 2 h, 4 h, or 12 h after the UVB exposure, depending on the assay. Each parameter was evaluated at a specific time point, which was previously determined as suitable to detect significant differences between negative and positive control groups, therefore, being valid for determination of possible treatment effect^10–12,57^. Experiments were conducted twice.

### Irradiation protocol

UVB lamp (Philips TL/12 RS 40 W, Medical-Holand) emits light between 270 and 400 nm, peaking at 313 nm, and placed on the top of the irradiation chamber and positioned 20 cm above the mice. This distance results in an irradiation of 0.384 mW/cm^2^. The radiation dose for induction of inflammation and oxidative stress was 4.14 J/cm^1,10–12,32^. Specific time points after the UVB exposure were used based on previous standardization protocols^10–12,36^.

### Skin edema

Dorsal skin biopsy was carefully removed from euthanized mice and weighed using a precision scale. All samples presented a constant diameter of 5 mm. Results are expressed in mg of skin tissue obtained from the weight of each sample^10,11^.

### MPO activity assay

MPO colorimetric assay was used to determine neutrophil migration to the skin^10,27^. Reading was performed at 450 nm. A standard curve of neutrophils was used to compare the results, which are presented as MPO activity (number of neutrophils x 10^4^ per mg of skin).

### Skin fluorescence

Shaved LysM-eGFP^+^ mice were used to visualize leukocyte recruitment to the skin as described previously^11^. This report mouse strain expresses enhanced green fluorescent protein (eGFP) expression controlled by the lysozyme M promoter (LysM) present in neutrophils granules. Imaging was performed using a confocal microscope (Leica TCS SP8, Leica, Wetzlar, Germany) with a 20x long distance objective. Images were processed using Leica EL6000 software (Leica, Wetzlar, Germany). The intensity of fluorescence was quantified by an investigator blinded to the treatment in randomly selected fields (one field per sample, n = 5) of different groups as an indicative of neutrophils recruitment to the skin tissue. The results are expressed as eGFP fluorescence intensity (%)^11^.

### Histopathological analysis

Skin samples were fixed in buffered formaldehyde, embedded in paraffin, sectioned (5 mm), and stained with Masson’s trichrome stain for collagen fiber analysis. Collagen fiber intensity bundles shown in blue were analyzed by ImageJ Program as described previously^11^. Tissue sections were also stained with hematoxylin and eosin (H&E), and images were analyzed for epidermal thickness and apoptotic cells^11^. Toluidine blue staining was also used to determine mast cells count^35^. Histopathological scores are presented together with the representative images quantifying the alterations detected between the groups.

### Cytokine measurement

The levels of skin IL-1β, TNFα and IL-10 were measured using commercial enzyme-linked immunosorbent assay (ELISA) kits according to manufacturer’s instructions (eBioscience)^11^. For that, skin samples were dissected and homogenized into sterile saline (500 µL). The supernatant was used to determine cytokine levels. Reading was performed at 450 nm in a microplate spectrophotometer reader and the results are expressed as picograms (pg) of each cytokine/mg of skin tissue.

### Real time and quantitative polymerase chain reaction (RT-qPCR)

Skin samples were dissected into TRIzol reagent (Invitrogen) and total RNA was extracted as recommended by manufacturer. The ratio of the reading at 260 and 280 nm was used to determine RNA purity (between 1.8 and 2.0 for all preparations). Reverse transcription of total RNA to cDNA and qPCR were performed using GoTaq® 2-Step RT-qPCR System (Promega, Madison, WI, USA) on a StepOnePlus™ Real-Time PCR System (Applied Biosystems®, Thermo Fisher Scientific, Waltham, MA, USA). The relative gene expression was determined using the comparative 2^−(∆∆Ct)^ method and GAPDH mRNA expression was used a reference gene to normalize data. Primer sequences were described previously^11^.

### Matrix metalloproteinase (MMP)-9 activity measurement

SDS-PAGE (sodium dodecyl sulphate polyacrylamide gel electrophoresis) substrate-embedded enzymography was performed as described previously^11^. After electrophoresis, the gels were incubated with 2.5% Triton X-100 (1 h), with 0.05 M Tris-HCl (pH 7.4), 0.01 M CaCl_2_ (overnight, 37 °C), and stained with Brilliant blue R. The zone of enzyme activity was analyzed by comparing the groups in the ImageJ Program, after detaining in 20% acetic acid (NIH, Bethesda, MD, USA). A total of 4 gels were analyzed and each one presented the results of pools of 6 mice per group.

### GSH assay

GSH levels were determined as described previously^11,36^. Reading was performed at 405 nm. Results are presented as μM of GSH per mg of skin, compared to standard curve (GSH concentration ranging 5–150 μM).

### Catalase activity assay

To determine catalase activity, it was measured the decay on H_2_O_2_ concentration and the oxygen generation^11,36^. Reading was performed at 240 nm (25 °C) and catalase activity was calculated based on the difference between the reading before and 30 sec after H_2_O_2_. The catalase values were expressed as unit of catalase/mg of skin/minute.

### Total antioxidant capacity: ABTS and FRAP assays

For both assays, skin samples were dissected, homogenized into ice-cold buffer containing 1.15% KCl, centrifuged (1,000 g in 4 °C for 10 min), and antioxidant capacity was determined as described previously^11,58^. Reading was performed at 734 nm and at 595 nm respectively. All results were compared to a standard curve of trolox (concentration ranging between 0.01–20 nmol). Results are presented as nmol trolox equivalent per mg of skin tissue.

### Superoxide anion production

Superoxide anion (O_2_^•−^) production in the skin was measured using the nitroblue tetrazolium (NBT) reagent as described previously^10,11^. Reading was performed at 620 nm. Results are expressed as NBT reduction (OD/ 10 mg of skin).

### Lipid peroxidation assay

Lipid peroxidation was measured by tert-butyl lipid hydroperoxides (LOOH)-initiated chemiluminescence^11,36^. Reading was conducted in a β-counter Beckman®LS 6000SC in a non-coincident counting for 30 s with a response range between 300 and 620 nm. All runs last 120 min (30 °C). The results were measured in counts per min (cpm) per mg of skin tissue.

### Data analysis

Results are presented as mean values ± standard error of the mean (SEM) and representative of two independent experiments. Each experimental group presented 5 LysM-eGFP mice or 6 hairless mice per experiment. Statistical analysis was performed on the software GraphPad Prism 6 (GraphPad Software Inc., San Diego, CA, USA) using one-way ANOVA followed by Tukey’s *post-hoc*. When *p* < 0.05, results were taken as statistically significant.

## Supplementary information

Supplementary Figure S1

## Data Availability

All data are presented in the manuscript. Additional information will be provided upon request.
