# Supplementary material for: Treatment with maresin 1, a docosahexaenoic acid-derived pro-resolution lipid, protects skin from inflammation and oxidative stress caused by UVB irradiation
Source: Sci Rep. 2019 Feb 28;9:3062. doi: 10.1038/s41598-019-39584-6 (PMC6395735; doi:10.1038/s41598-019-39584-6)
Supplement: Supplementary file 1 — Supplementary Figure S1 [file 41598_2019_39584_MOESM1_ESM.pdf]

Treatment with maresin 1, a docosahexaenoic acid-derived pro-resolution lipid, protects skin from inflammation and oxidative stress caused by UVB irradiation

Talita L. C. Cezar[1], Renata M. Martinez[1], Camila da Rocha[1], Cristina P. B. Melo[1], David L. Vale[1], Sergio M. Borghi[2], Victor Fattori[2], Josiane A. Vignoli[3], Doumit Camilios-Neto[3], Marcela M. Baracat[1], Sandra R. Georgetti[1], Waldiceu A. Verri, Jr[2], Rúbia Casagrande[1]*

[1]Laboratory of Oxidative Stress and Inflammation, Department of Pharmaceutical Sciences, Londrina State University, Londrina, PR, Brazil.

[2]Laboratory of Pain, Inflammation, Neuropathy, and Cancer, Department of Pathology, Londrina State University, Londrina, PR, Brazil.

[3]Department of Biochemistry and Biotechnology, Londrina State University, Londrina, PR, Brazil.

*Corresponding author. Adress: Avenida Robert Koch, 60, Vila Operária, CEP 86039-440 Londrina, Paraná, Brazil. Tel.: +55 43 33712475. E-mail address: rubiacasa@yahoo.com.br (R. Casagrande).

Supplementary figure S1

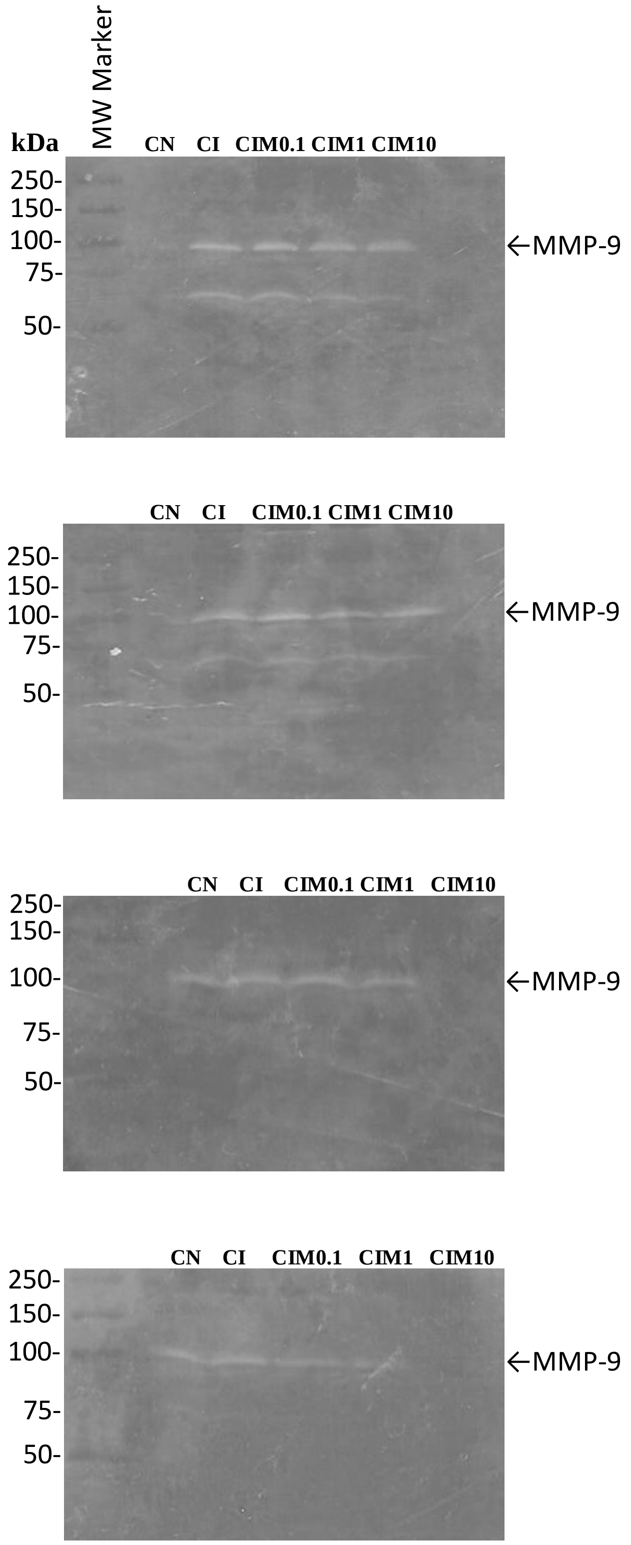

Supplementary Figure S1. Uncroped full-length gel membranes of gelatin zymography from which the representative image shown in figure 5 in the main manuscript was croped. Each sample is a pool of the dorsal skin of 6 hairless mice. CNI=control non-irradiated; CI=control irradiated treated with vehicle; CIM0.1=control

irradiated treated with 0.1 ng of MaR1; CIM1=control irradiated treated with 1 ng of MaR1;and CIM10=control irradiated treated with 10 ng of MaR1.
